# Factors Related to the Development of Infective Endocarditis in Hemodialysis Patients in a Third-Level Hospital in Panama

**DOI:** 10.7759/cureus.52385

**Published:** 2024-01-16

**Authors:** Harold A Bravo Thompson, Francisco A Campos Herrera, David A Macías Ibiricu, Sara I Rodríguez Barrios, Daniella I Vázquez Acevedo, Chantal A Candanedo Gonzalez, Grisel López, Ricardo Gollini

**Affiliations:** 1 Internal Medicine, University of Miami Miller School of Medicine, Jackson Memorial Hospital, Miami, USA; 2 General Surgery, Complejo Hospitalario Arnulfo Arias Madrid, Panama City, PAN; 3 Medicine, University of Panama, Panama City, PAN; 4 General Surgery, Hospital Santo Tomás, Panama City, PAN; 5 Emergency Medicine, Kendall Regional Medical Center, Miami, USA

**Keywords:** central venous catheters, vascular access devices, chronic kidney disease, hemodialysis, bacterial endocarditis

## Abstract

Introduction: Venous access for hemodialysis (HD) makes patients more susceptible to transient bacteremia, predisposing them to the development of infective endocarditis (IE). Among the risk factors observed in this population are temporary access to HD, hypoalbuminemia, diabetes mellitus, female gender, anemia, and colonization by methicillin-resistant *Staphylococcus aureus* (MRSA).

Methodology: A retrospective case-control study with a one-to-two ratio was carried out on patients with chronic kidney disease (CKD) undergoing renal replacement therapy with at least one vascular access for HD at Complejo Hospitalario Dr. Arnulfo Arias Madrid (CHDrAAM) from 2010 to 2020. Sociodemographic variables, past medical history, and data on current HD were studied. The odds ratio (OR) and adjusted odds ratio (aOR) were calculated for the collected variables.

Results: No statistically significant differences between the groups were observed in sociodemographic variables. In terms of past medical history, the cases showed a predominance of coronary disease (47.6% vs 4.8%; OR: 37.27), valvular disease (23.8% vs 0%), and heart failure (33.3% vs 4.8%; OR: 10). In the cases, the use of a temporary catheter was more prevalent (61.9% vs 33.3%; OR: 3.25), and subclavian access was more frequently recorded (28.6% vs 2.4%; OR: 14.4). A short duration of venous access (<30 days) was found in a greater proportion of cases (23.8% vs 4.8%; OR: 6.25). The main pathogen isolated was S. aureus (33.3%), and the most affected valve was the aortic valve (59.1%). Fever was found in 100% of the reported cases, and up to 47.6% presented with a recent murmur.

Discussion: Similar to previous studies conducted in other countries, we identified a history of pre-existing valve disease, the use of a temporary catheter, and recent venous access as risk factors. Contrary to what has been reported in the literature, this study did not find female sex, diabetes mellitus, and hypoalbuminemia as risks.

Conclusion: Factors such as a history of coronary artery disease, heart failure, preexisting valvular disease, the use of a temporary catheter, subclavian venous access, and short duration of venous access (<30 days) were identified as risk factors for the development of IE in patients with CKD on HD.

## Introduction

Infective endocarditis (IE) is the colonization of cardiac valves by an infectious agent circulating in the blood and adhering to the injured endothelium. The development of endocarditis requires the interaction of three simultaneous processes: susceptible endothelium, transient bacteremia, and microorganism invasiveness [1].

Venous access makes these patients more susceptible to transient bacteremia, compounded by a deficient immune system due to the lability of uremia, creating an environment conducive to colonization [1]. Bacteremia is the second leading cause of death in hemodialysis (HD) patients, after cardiovascular diseases [2]. In Panama, according to the National Dialysis Coordination of the Social Security Fund, there are 2,714 patients with chronic renal disease on HD and only 17 HD units [3,4,5].

IE is a rare disease with an incidence in developed countries of 3.1-7.9 per 100,000 inhabitants, while in HD patients, this incidence increases to 267-308 per 100,000 inhabitants [6,7]. Thus, the risk is 16.9-18 times higher than in the population without renal replacement therapy [2,6,8]. Although data from Panama are lacking, information from Europe and the United States indicates an increasing incidence in recent years despite the increased use of antibiotics. This rise is attributed to a higher incidence of degenerative valve disease with subsequent valve replacement, valve calcification, invasive procedures with repeated bacteremia, increased consumption of intravenous drugs, and immune deficiencies related to uremia [2,6,9].

Among the risk factors, literature cites HD access type, with the catheter being the main risk factor [2,7,10], hypoalbuminemia, diabetes mellitus, ischemic heart disease, underlying heart failure, peripheral vascular disease, cerebrovascular disease, anemia [10], female sex, and colonization by methicillin-resistant *Staphylococcus aureus* (MRSA) [11]. The clinical presentation tends to be more silent and chronic, usually presenting with fever, weight loss, excessive sweating, anemia, adynamia, and in some cases Osler nodules, Janeway lesions, and Roth's spots, in addition to a de novo heart murmur [12].

The modified Duke criteria are used for diagnosis. The combination of two major criteria or one major criterion and three minor criteria or five minor criteria offers a specificity close to 99% [13]. Once IE is diagnosed, a patient's prognosis is significantly impoverished by a significant percentage, with in-hospital mortality of 23.3% and one-year mortality of 61.6% [6]. These figures can increase in the context of the Latin American health system to an estimated 75% in a study conducted in Chile [14]. Considering that a patient undergoes approximately three weekly sessions, and each session costs around 200 dollars according to the Santo Tomas Hospital, the monthly HD of one patient impacts the Panamanian public system with around $2,600 per month [15]. According to a study in the United States, hospitalization costs for patients with infectious endocarditis are close to one million dollars and are increasing due to the growing number of patients in HD units annually [16].

The objective of this research was to determine the risk factors related to the development of IE in chronic HD patients at Complejo Hospitalario Dr. Arnulfo Arias Madrid (CHDrAAM) between 2010 and 2020, in addition to describing sociodemographic variables, the most common microorganism, and clinical features. The study also details the most affected cardiac valves and laboratory values before therapy.

This article was previously presented as a poster at the 2023 American College of Physicians Internal Medicine Meeting on April 27, 2023.

## Materials and methods

An observational, analytical, retrospective case-control, unpaired 1:2 study was conducted on patients on RRT with at least one vascular access (HD) at the CHDrAAM from 2010 to 2020, including those who develop IE (cases) and those who do not (controls) according to the modified Duke criteria for diagnosis of IE. Simple random sampling was performed after coding the charts.

Patients older than 18 years with a diagnosis of IE recorded in the chart or with a Duke criteria record supporting the diagnosis of IE, who were on RRT with at least one vascular access, were included. The control group included patients (older than 18 years) who were on RRT with at least one vascular access, with no diagnosis of IE in the record and no record of modified Duke criteria supporting the diagnosis of IE. Files with less than 80% of the information required in the clinical record, with a diagnosis of IE before RRT, with acute renal failure requiring acute HD, and those with only conservative measures, peritoneal dialysis, and pregnant women were excluded.

Demographic variables of sex, age, and BMI were collected. Also, past medical history such as valvular disease, arterial hypertension (HTN), type 2 diabetes mellitus (DM2), congestive heart failure (CHF), coronary artery disease (CAD), human immunodeficiency virus-positive (HIV+) and immunosuppressive medication. Finally, their current RRT data were evaluated including age at HD initiation, duration of HD until IE diagnosis, type of venous access, location of venous access, duration of venous access, and number of previous venous accesses.

The sample calculated to achieve statistical power was 10 cases and 20 controls with the Epi-Info 7.2.1 program with the Fleiss formula with a correction factor for unpaired cases and controls, using the odds ratio (OR) of 2.65 and a percentage of exposed controls of 72.84 % from the study by Fram et al. [17] with HTN as a risk factor, with 95% confidence level, power of 80%. A total of 21 cases and 42 controls (total n=63) were obtained.

Data were tabulated using Microsoft Excel 2013 (Microsoft Corporation, Redmond, Washington). For the statistical analysis, the Epi Info 7.2.1 program was used. A bivariate Pearson's X^2^ analysis was performed for qualitative variables and T-Student for quantitative variables. Significant variables were subjected to multivariate logistic regression. The strength of association was established by calculating OR and adjusted odds ratio (aOR) for possible confounding variables. They will be taken as significant based on their 95%CI.

The study was approved by the Institutional Ethics Committee of the University of Panama and permissions for the review of clinical records from the CHDrAAM were granted through note C.H.Dr,A.A.A.M./SdeN/049/2019. Considering that the research methodology involves low risk for the participants as it is a file review, the bioethics committee did not require the request for informed consent. Confidentiality was maintained as no data were collected that would allow their identification.

## Results

Among the sociodemographic variables, a predominance of male sex was observed in both groups (76.2% in cases vs 73.8% in controls). The mean age was 59.4±11.3 years for cases and 56.8±13.8 years for controls. As for weight before HD, there was wide variability (71.8±15.5 kg in cases vs 75.3±15.9 kg in controls).

Regarding personal pathological history (PPH), more than 80% of the population studied had HT (85.7% in cases vs 83.3% in controls, respectively). The case group showed a greater predominance of coronary heart disease (47.6% vs 4.8%), heart failure (33.3% vs 4.8%), and previous valvular disease (23.8% vs 0%) compared to controls. The rest of the personal pathological history, including DM2, HIV seropositivity, and use of immunosuppressive medication, is presented in Table 1.

**Table 1:** Baseline characteristics of the study population (n = 63) Values are presented as mean ± SD or absolute frequency and percentages. P-value significant at <0.05. NC: non-quantified, M: male, F: female, y: years, HTN: arterial hypertension, DM2: diabetes mellitus type 2, HIV: human immunodeficiency virus, IE: infective endocarditis, HD: hemodialysis, AVF: arteriovenous fistula, CVC: central venous catheter. Source: Study database. Complejo Hospitalario Dr. Arnulfo Arias Madrid 2010-2020

Characteristics	Cases, n = 21	Control, n = 42	p
Sociodemographic characteristics			
Sex – male	76.2%	73.8%	0.547
Sex – female	23.8%	26.2%	0.547
Age	59.4 ± 11.3 y	56.8 ± 13.8 y	0.4475
Weight	71.8± 15.5 kg	75.3 ± 15.9 kg	0.9475
Personal pathological history			
HTN	85.7% (18)	83.3% (35)	0.559
Coronary artery disease	47.6% (10)	2.4% (1)	0.00002
Heart failure	33.3% (7)	4.8% (2)	0.0046
Valvular disease	23.8% (5)	0%	0.0029
DM2	38.1% (8)	47.6% (20)	0.328
HIV-positive	4.8% (1)	9.52% (4)	0.455
Immunosuppressant medication	4.8% (1)	11.9% (5)	0.340
Current hemodialysis (at diagnosis of IE)			
Age of HD onset	56.5 ± 11.6 y	55.1 ± 13.9 y	0.7125
Duration of HD until diagnosis of IE 0–30 days	4.8% (1)	NC	NC
Duration of HD until diagnosis of IE 30–180 days	38.1% (8)	NC	NC
Duration of HD until diagnosis of IE >180 days	57.1% (12)	NC	NC
Type of venous access			
AVF	0%	35.7% (15)	0.0008
Tunneled CVC	38.1% (8)	31.0% (13)	0.385
Non-tunneled CVC (temporal)	61.9% (13)	33.3% (14)	0.029
Vascular access location			
Upper extremity	14.3% (3)	35.7% (15)	0.034
Jugular vein	47.6% (10)	45.2% (19)	0.500
Subclavius vein	28.6% (6)	2.4% (1)	0.0070
Femoral vein	9.5% (2)	4.8% (2)	0.460
NC	0%	11.9% (5)	---
Vascular access duration			
0–30 days	23.8% (5)	4.8% (2)	0.036
30–180 days	38.1% (8)	23.8% (10)	0.187
>180 days	38.1(8)	71.4% (30)	0.011
Number of prior venous accesses 0	28.6% (6)	19.0% (8)	0.191
Number of prior venous accesses 1–2	42.8% (9)	50.0% (21)	0.610
Number of prior venous accesses >2	14.3% (3)	31.0% (13)	0.206
Number of prior venous accesses NC	14.3% (3)	0%	NC

Regarding HD parameters, the mean age at which patients entered the HD program included a working-age population (56.5 ± 11.6 years for cases vs. 55.1 ± 13.9 years for controls). More than half of the cases were documented >180 days after starting HD (57.1%) (Table 1).

Regarding the type of venous access, the group of cases was more prevalent with the use of a transient catheter (61.9%), and none of the cases had AVF as venous access. Regarding the location of the vascular access, the cases had more frequent jugular access (47.6%) and subclavian (28.6%) vs. jugular (45.2%) and MSI (35.7%) in the controls. Regarding the number of previous accesses, almost half of both groups already had one to two previous accesses at the time of inclusion (42.8% and 50.0% for cases and controls, respectively) (Table 1).

Description of IE cases

**Figure 1:**
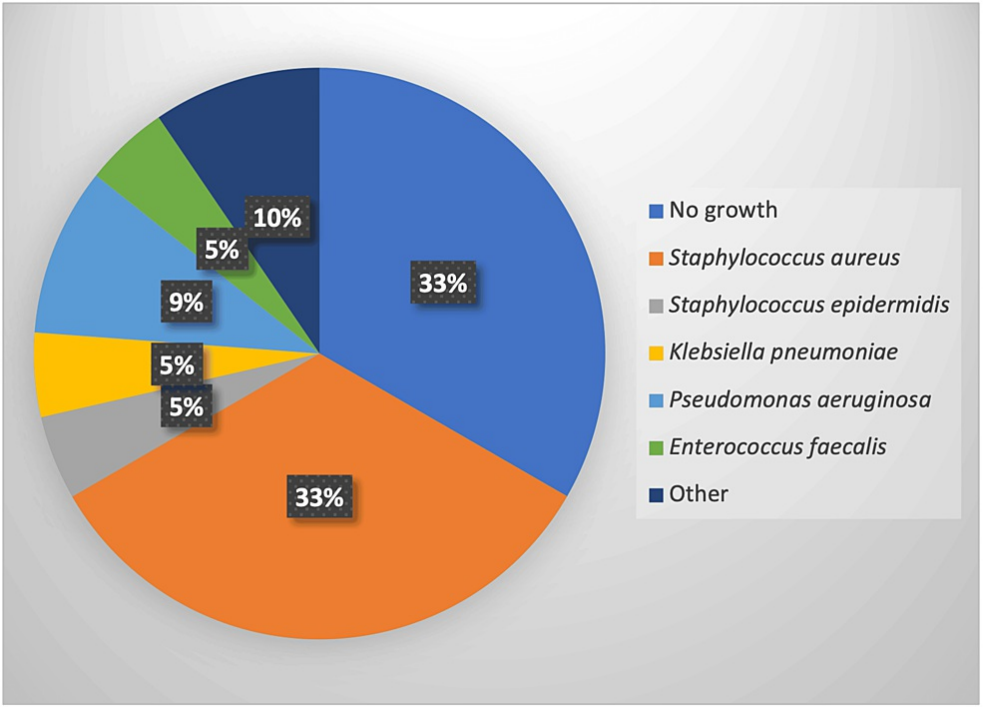
Isolated microorganism Source: Study database. Complejo Hospitalario Dr. Arnulfo Arias Madrid 2010-2020

Regarding microbiological isolation, in 33.3% of cases, no microorganism was isolated. Another third was caused by Staphylococcus aureus at 33.3% (7 cases), and the rest by varied microbiology: *Staphylococcus epidermidis* 4.76% (7 cases), *Klebsiella pneumoniae *4.76% (1 case), *Pseudomonas aeruginosa *9.52% (2 cases), *Enterobacter cloacae *4.76% (1 case), *Enterococcus faecalis *4.76% (1 case), and *Elizabethkingia meningoseptica *4.76% (1 case) (Figure 1).

All cases reported fever (21) and up to 47.6% (10 cases) had a new onset murmur; followed by weakness and/or fatigue in 71.4% (15 cases), dyspnea in 66.7% (14 cases), and weight loss in 4.76% (1 case). Vascular phenomena, including major arterial embolism, septic lung infarction, infectious (mycotic) aneurysm, conjunctival hemorrhage, and Janeway lesions, were recorded in only one case, while immunological phenomena, including splinter hemorrhages, glomerulonephritis, Osler's nodules, and Roth's spots, were not recorded in this case series.

The most affected valve was the aortic valve in 59.1% (13 cases), followed by the mitral valve in 31.8% (7 cases), and finally the tricuspid valve in 9.1% (2 cases). Most of the patients were admitted with leukocytosis (87.7%) with a variable average (15,909 ± 6,734 cells/mm³), without evident thrombocytopenia (203,309 ± 96,980 cells/mm³) and with a tendency to hypoalbuminemia (3.09 ± 0.61 mg/dL).

Risk factors found

The risk estimates for the variables studied are summarized in Table 2.

**Table 2:** Risk estimation of variables studied P value significant at <0.05. CI: confidence interval. ND: non-defined.  HTN: arterial hypertension, DM2: diabetes mellitus type 2, HIV: human immunodeficiency virus, IE: infective endocarditis, HD: hemodialysis, AVF: arteriovenous fistula, CVC: central venous catheter. Source: Study database. Complejo Hospitalario Dr. Arnulfo Arias Madrid 2010-2020

Variables	OR (95%CI)	Logistic regression (p)
Sociodemographic characteristics		
Sex	1.13 (0.33-3.83)	-
Age	P=0.4475	0.366
Weight	P=0.9475	0.164
Personal pathological history		
HTN	1.20 (0.27-5.20)	0.304
Coronary artery disease	37.27 (4.29-323.43)	0.003
Heart failure	10.00 (1.85-53.93)	0.855
Valvular disease	ND	0.007
DM2	0.67 (0.23-1.97)	0.829
HIV positive	0.47 (0.05-4.54)	0.960
Immunosuppressant medication	0.37 (0.04-3.39)	0.290
Current hemodialysis (at diagnosis of IE)		
Age of HD onset	P=0.7125	0.412
Type of venous access		
AVF	ND	0.0056
Tunneled CVC	1.37 (0.46-4.11)	0.0056
Non-tunneled CVC (temporal)	3.25 (1.09-9.66)	0.0056
Vascular access location		
Upper extremity	0.24 (0.06-0.979)	0.0092
Jugular vein	0.86 (0.29-2.52)	0.0092
Subclavius vein	14.40 (1.59-130.10)	0.0092
Femoral vein	1.84 (0.24-14.13)	0.0092
Vascular access duration		
0-30 days	6.25 (1.10-35.58)	0.0173
30-180 days	1.96 (0.63-6.10)	0.0173
>180 days	0.25 (0.08-0.74)	0.0173
Number of prior venous accesses		
0	2.12 (0.61-7.39)	0.3803
1-2	1.00 (0.33-3.02)	0.3803
>2	0.44 (0.07-2.02)	0.3803

**Figure 2:**
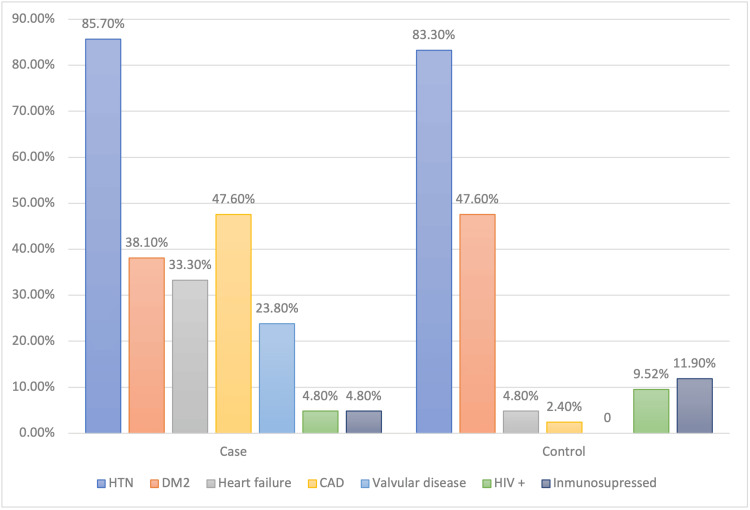
Personal pathological history HTN: arterial hypertension, DM2: diabetes mellitus type 2, CAD: coronary artery disease, HIV+: human immunodeficiency virus-positive. Source: Study database. Complejo Hospitalario Dr. Arnulfo Arias Madrid 2010-2020

We found an increased risk of IE in patients with certain comorbidities: coronary artery disease (37 times), heart failure (10 times), and previous valvular disease (OR not calculable due to small sample size) (Figure 2).

**Figure 3:**
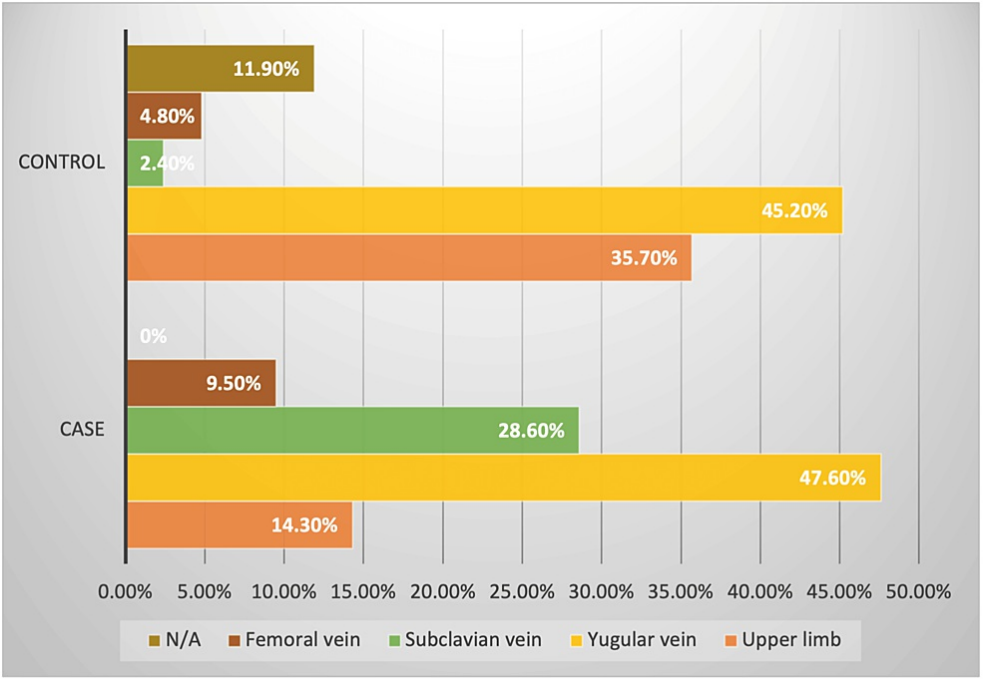
Vascular access location N/A: not applicable or not defined. Source: Study database. Complejo Hospitalario Dr. Arnulfo Arias Madrid 2010-2020

Regarding the main variables of interest, which were HD parameters, statistically significant differences were found according to the type of venous access used, its location, and duration. Having a non-tunneled central venous catheter carries a 3.25-fold risk of contracting endocarditis, as does placement in the subclavian area (14.4 times). However, placement in the upper limb was associated with a 76% decrease in risk, possibly associated with the fact that venous accesses for HD in the upper limb are usually tunneled or are arteriovenous fistulas (AVFs) (Figure 3).

Concerning temporality, we found the first 30 days of venous access placement to be the period of greatest risk for the development of IE, and although counterintuitive, we found a 75% reduction in the risk of IE if the venous access lasted more than 180 days.

## Discussion

This study provides additional information to that previously reported in the literature and offers starting points to justify clinical trials or more rigorous studies to demonstrate and modify the risk determinants found.

Studies in the literature agree that the most frequently isolated microorganism in cases of IE in HD patients is *S. aureus* and other coagulase-negative staphylococci such as *S. epidermidis *[2,3,6,7,10,16-19], representing almost 50% of the isolates, as corroborated in our study. This indicates that the most frequent port of bacteremia in these patients corresponds to the vascular access site [6,7,18].

On the other hand, Enterococcus is the second cause of IE in this population, and a gradual increase has been observed due to the increase in healthcare-related cases and the growing complexity of the patients in terms of comorbidities [10,16,20,21]. This does not align with our results, possibly due to the small number of cases. However, various cohorts agree that Enterococcus is much more common as a cause of IE in the general population [10,18]. Other authors found a higher frequency of polymicrobial IE in HD patients [10], and, like us, a significant number of negative cultures (>25%) [14,17]. Regarding the culture-negative cases, the hospital where the study was conducted does not have an institutional policy on serology or additional advanced culture workup, thus the decision to obtain further workup was at the discretion of the treating physician. In the cases recorded, the investigators were not able to find any additional negative or positive workup documented. We hypothesize that some negative cases can be explained by some of the patients receiving antibiotics before the blood cultures were taken, therefore obscuring some of the results. However, these patients still fulfilled the Duke criteria by other means.

Regarding the clinical presentation of IE in HD patients, our cases are consistent with those reported by other authors, the most common being fever (>70%), new onset murmur (>75%), and dyspnea (>30%) [16,22,23]. Weight loss and fatigue (>60%), lower limb edema (>30%), cough (20%), chest pain (15%), and arthralgias/myalgias (12%) are also reported. In terms of complementary studies, Romani et al. agree with our series in leukocytosis (>10,000) without thrombocytopenia and with a similar tendency to hypoalbuminemia (mean close to 3.0 g/dL) [12].

The most affected valve differs in the different studies, being in most of the series the mitral valve [10,16,24], while in others, such as ours, it is the aortic valve [12,14].

In large cohorts such as that carried out by Pericas et al [10], the female sex is predominant in HD patients, while our population shows a predominance of the male sex, as do other authors [2,6,12,22]. However, as regards sex, we did not find it to be a risk factor, as reported in the conflicting literature [7,16,19].

The median age is variable in the studies from 48.3 to 69.7 but they are like that studied in our population of 59 years [2,6,10,12,16,22]. A Danish cohort study surprisingly found a decreased risk of endocarditis in those older than 70 years, but this could be due to selection bias in this population as they may be too frail for a transesophageal echocardiogram or have died before the diagnosis of IE [18].

HD patients with IE present with high percentages of cardiovascular risk factors and their complications such as diabetes mellitus, hypertension (HT), baseline heart failure, peripheral arterial disease, cerebrovascular disease, and ischemic coronary artery disease [6,10,14,17]. This is in line with the known epidemiology of the chronic renal patient on HD, who is at intrinsically higher risk of accelerated atherosclerotic disease [25]. In our data, we found statistical significance for congestive heart failure (CHF), coronary artery disease, and previous valvular disease in agreement with the study by Chaudry et al in a Danish population [7]. However, the low number of cases may influence the decrease in statistical power and therefore not reach statistical significance. 

A cross-sectional study in the United States found a higher prevalence of HIV infection and AIDS stage among dialysis patients; however, as well as the use of immunosuppressive medication and the presence of malignant neoplasms, this has not been shown to be a risk factor for the development of IE in the HD population [6,10].

Regarding HD variables, we found, as reported in other studies, that the greatest risk factor is derived from the transient catheter, with graft and arteriovenous fistula (AVF) being the lowest risk. Transient HD catheters have the highest incidence of infection, bacteremia, and subsequent IE compared to both native AVF and AV graft (with up to 14 times the risk of endocarditis) [6,7,10,17,18,23,26]. In this study, none of the cases of IE occurred in patients with AVFs, coinciding with its finding as a protective factor for IE in large national cohorts [7,18]. The Fistula First Initiative includes a series of guidelines on HD vascular accesses recommending fistulas or AV grafts as the preferred modality and a goal of having less than 10% of HD patients with tunneled central venous catheters (CVC). Even more, if possible, to avoid the use of non-tunneled CVC [27].

Recent studies also show that recently placed AVFs can be a potential port of entry for IE, with cases on the rise [10]. This may be related to repetitive vascular puncture during HD and is supported by the fact that most of these reported cases were caused by a commensal skin mycobacterium [7]. Given that in our study none of the cases reported were in patients with AVFs, it is impossible to make any assertion in this regard. However, it must be considered when evaluating symptomatic patients in HD units, and they should not be overlooked as potential sources of bacteremia in these patients.

Concerning location, we found that both femoral and subclavian access confer a greater risk for endocarditis. Herazo et al. found significant differences in the frequency of HD catheter infections in the Colombian population, with jugular site placement as a protective factor. Given that HD catheter infection may precede the development of IE, it is reasonable that the results obtained are in the same direction [19].

Some cohorts [7,17,18] found recent vascular access placement (within the first six months) to be a risk factor for the development of bacteremia and IE, which correlates with our results of a 5.35 times increased risk of endocarditis during the 30 days after vascular access placement. These findings reinforce the need to maximize hand hygiene and sterility measures in the placement of these vascular accesses and, preferably, to perform them in a controlled environment such as the operating room, to reduce the potential bacteremia in this period.

Limitations and strengths

IE corresponds to a rare diagnosis, even in patients with chronic kidney disease (CKD) undergoing HD, so the number of patients included in this study is small and may be subject to potential selection bias [12,23]. In addition, regarding the culture-negative cases, the hospital where the study was conducted does not have an institutional policy for additional workup, and some negative cases can be explained by some of the patients receiving antibiotics before the blood cultures were taken, therefore obscuring some of the results.

Nevertheless, this study represents a series obtained from the main urban hospital in Panama City and therefore constitutes an adequate representation of Panamanian epidemiology. Furthermore, it represents one of the few published studies to specifically investigate associated risk factors for the development of IE in this population, beyond HD catheter infection and bacteremia.

## Conclusions

This publication accurately depicts the risk factors associated with the development of IE in patients with CKD receiving HD beyond HD catheter infections or bacteremia. We found as a risk factor a history of coronary artery disease, heart failure, or preexisting valvular disease, which justifies more rigorous surveillance in this type of patient and a low index of suspicion and treatment for IE in this population.

Additionally, the results suggest that the use of a transient catheter, especially subclavian venous access, during the first 30 days of catheter placement, confers an increased risk of developing endocarditis. This, together with the enormous cost to the health system generated by an episode of endocarditis in this population, justifies the development of policies to migrate from the use of transient catheters to AVFs and to strengthen hand washing campaigns and proper care of HD access catheters.
